# Expression of TAM-R in Human Immune Cells and Unique Regulatory Function of MerTK in IL-10 Production by Tolerogenic DC

**DOI:** 10.3389/fimmu.2020.564133

**Published:** 2020-09-25

**Authors:** Paul Giroud, Sarah Renaudineau, Laura Gudefin, Alexandre Calcei, Thierry Menguy, Caroline Rozan, Jacques Mizrahi, Christophe Caux, Vanessa Duong, Jenny Valladeau-Guilemond

**Affiliations:** ^1^Elsalys Biotech SA, Lyon, France; ^2^Université Claude Bernard Lyon 1, INSERM U1052 CNRS 5286, Centre Léon Bérard, Centre de Recherche en Cancérologie de Lyon, Lyon, France

**Keywords:** MerTK, dendritic cells, macrophages, tumor immunity, immunosuppression

## Abstract

Tumor-infiltrating myeloid cells are a key component of the immune infiltrate often correlated with a poor prognosis due to their capacities to sustain an immunosuppressive environment. Among membrane receptors implicated in myeloid cell functions, Tyro3, Axl, and MerTK, which are a family of tyrosine kinase receptors (TAM-R), have been described in the regulation of innate cell functions. Here, we have identified MerTK among TAM-R as the major marker of both human M2 macrophages and tolerogenic dendritic cells (DC). *In situ*, MerTK expression was found within the immune infiltrate in multiple solid tumors, highlighting its potential role in cancer immunity. TAM-R ligands Gas6 and PROS1 were found to be constitutively produced by myeloid cells *in vitro*. Importantly, we describe a novel function of MerTK/PROS1 axis in the regulation of IL-10 production by tolerogenic DC. Finally, the analysis of TAM-R expression within the lymphoid compartment following activation revealed that MerTK, but not Axl or Tyro3, is expressed on activated B lymphocytes and regulatory T cells, as well as CD4^+^ and CD8^+^ T cells. Thus, our findings deepen the implication of MerTK in the regulation of myeloid cell-mediated immunosuppression and identified new cellular targets expressing MerTK that could participate in the antitumor immune response.

## Introduction

The tumor microenvironment (TME) of many cancer cell types is characterized by a high infiltration of myeloid cells. These tumor-associated myeloid cells—which constitute a heterogenous population including monocytes, macrophages, dendritic cells, and granulocytes—are considered as relevant therapeutic targets. They share functional features with suppressive activity that enable strong immunosuppression and favor tumor cell proliferation and progression. Among myeloid cells, macrophages represent a significant portion of immune infiltrate in most cancers and have been linked to poor prognosis (1). Macrophages exhibit extensive functional plasticity dependent on activation or microenvironmental cues (2). Classically, macrophage phenotypes are simplified by opposing M1 pro-inflammatory macrophages, which are considered as anti-tumoral, to M2 immunosuppressive macrophages which are associated with poor prognosis (1). Dendritic cells are also major players in the TME, whose infiltration has been demonstrated in multiple indications (3). However, the prognostic implication of different subsets of dendritic cells (DC) in tumors remains unclear as they have been linked with both good and poor prognosis. Among DC subsets found in the TME, monocyte-derived DC (moDC) can display either an inflammatory or a tolerogenic phenotype (4). Immature moDC are characterized by a low expression of co-stimulatory molecules, production of immunosuppressive cytokines, and suppressive capacities toward T cells (5, 6). Thus, these cells are believed to participate in tumor-mediated immunosuppression.

Current therapeutic developments explore the potential of targeting the myeloid cell compartment. Notably, a family of receptor tyrosine kinases, Tyro3, Axl, and MerTK receptors (TAM-R) have been described as new regulators of myeloid immune response and as such represent promising therapeutic targets on tumor-associated myeloid cells.

TAM-R are essential for the homeostasis of the immune response. They contribute to homeostasis via their implication in the restoration of vascular integrity, the negative regulation of inflammation, and in the phagocytosis of dying cells, a process known as efferocytosis. The main TAM-R ligands are two vitamin K-dependent proteins: growth arrest specific 6 (Gas6) and protein S (PROS1). Gas6 binds to all members of the TAM-R family with a higher affinity for Axl, whereas PROS1 binds only to Tyro3 and MerTK, with a preference for the former (7). Gas6 and PROS1 share a similar structure with a N-terminus γ-carboxylated Gla domain, followed by four epidermal growth factor (EGF)-like repeats and two laminin G domains, that interacts with the two extracellular IgG-like domains of the TAM-R (8). Vitamin K-dependent γ-carboxylation of Gas6 and PROS1 is necessary for them to bind negatively charged phosphatidylserine residues exposed at the membrane of apoptotic cells, forming a bridge between the apoptotic membrane and the phagocyte expressing TAM-R (9). In macrophages, MerTK is essential to the efferocytosis of apoptotic cells opsonized by Gas6 or PROS1 (10).

TAM-R also function as negative regulators of inflammation. Mice lacking all three TAM-R develop a strong phenotype of autoimmunity and chronic inflammation as a result of the accumulation of cellular debris and hyperactivation of myeloid cells (11). Furthermore, genetic ablation of only MerTK in mice leads to an increase sensitivity to endotoxic shock in response to lipopolysaccharide (LPS), due to elevated levels of TNFα produced by macrophages, emphasizing MerTK essential role in resolution of inflammation (12). In DCs, Axl has been shown to be upregulated in presence of Toll-like receptors (TLR) ligands, and activation of Axl by Gas6 triggers the expression of suppressor of cytokine signaling (SOCS) 1 and 3, which in turn reduce the secretion of inflammatory cytokines (13, 14). More recently, tolerogenic moDC were shown to be able to suppress T cell response *in vitro* via a MerTK-dependent mechanism, underlining a role of MerTK at the interface between innate and adaptative immunity (15).

MerTK is widely described for its overexpression in solid and hematological tumors. MerTK activation promotes cell survival and migration and/or contributes to chemoresistance [reviewed in (16)]. Consistent with these functions, small-molecule inhibitors of TAM-R were developed demonstrating tumor growth inhibition in xenograft mouse models (17). MerTK also plays a prominent role in tumor progression through its expression in the tumor microenvironment. MerTK genetic ablation or inhibition by small molecules in leukocytes decreases tumor growth and enhances anti-tumor immune response by switching myeloid cell cytokine production from an immunosuppressive to an inflammatory state in an immunocompetent mouse model of breast cancer (18, 19). Furthermore, TAM-R have been shown to inhibit myeloid cell activation by reducing expression of costimulatory molecules or production of inflammatory cytokines in murine DC and macrophages (13, 20, 21).

To date, current knowledge of TAM-R expression in human immune cells remains partial (8). Recent reports of MerTK expression in activated T cells (15, 22) also call for investigations of TAM-R expression in the lymphoid compartment. To better understand the function of this receptor family, characterization of their expression as well as the production of their ligands is required. In human cells, however, it remains unclear if TAM-R signaling actively participates in immunosuppression.

In this study we analyzed MerTK, Tyro3, and Axl and their ligands Gas6 and PROS1 expression in human immune cells. We demonstrated that MerTK is expressed by immunosuppressive myeloid cells and activated T and B cells *in vitro*. Furthermore, we present evidence of MerTK expression in tumor-infiltrating immune cells in multiple cancer indications. Finally, we identified a novel regulation of IL-10 production in tolerogenic DC mediated by the PROS1/MerTK axis.

## Materials and Methods

### *In vitro* Myeloid Cells Differentiation

#### Generation of Human Macrophages

Human monocytes were isolated from leukoreduction system chambers from healthy donors (Etablissement Français du Sang, Lyon, France) by magnetic separation, using StraightFrom LRSC CD14 MicroBead Kit (Miltenyi Biotech) according to manufacturer's protocol. Purity of CD14^+^ cells was routinely 90–97%. CD14^+^ cells were seeded in 48- or 24-well plates at 1 · 10^6^ cells/ml for 7 days at 37°C in 5% CO_2_ in complete RPMI 1640 medium (Gibco) containing Glutamax™ and supplemented with 10% decomplemented fetal calf serum (FCS) and 40 μg/ml gentamicin. Cells were incubated from day 0 with GM-CSF (250 U/ml, Miltenyi) or M-CSF (4,000 U/ml, Miltenyi), to differentiate M1 or M2 macrophages, respectively. Medium and cytokines were refreshed at day 3. At day 5, IFNγ (500 U/ml, Miltenyi) was added in culture to achieve M1 differentiation. M2 macrophages were polarized with either IL-4 (400 U/ml, Miltenyi) or IL-10 (20 ng/ml, Miltenyi) and TGFβ (150 U/ml) to produce M2a or M2c macrophages, respectively. Tumor conditioned macrophages (TCM) were obtained by differentiation of CD14^+^ monocytes in 50% MDA-MB-231 conditioned medium. Briefly, MDA-MB-231 cells (ATCC) were cultured in T175 flask in DMEM medium (Gibco) containing 4.5 g/L D-glucose 1 mM pyruvate and supplemented with 10% FCS and 40 μg/ml gentamicin. Medium from confluent cells was replaced by fresh complete RPMI. After 30 h, conditioned medium was collected and stored at −80°C until use for TCM differentiation.

#### Generation of Human *in vitro* Derived DC

Monocytes from LRSC were isolated by gradient centrifugation with Ficoll-Paque Plus (GE Healthcare) and, subsequently, Percoll gradient separation (GE Healthcare). After 2 h at 37°C, non-adherent cells were removed, and complete medium containing 1,000 U/ml GM-CSF and 800 U/ml IL-4 (Miltenyi) was added. One micromole of dexamethasone (Dex) (Sigma) was added at day 3 or day 6 of differentiation to generate tolerogenic DC as described by Cabezón et al. (5). moDC phenotype at the end of differentiation was validated by flow cytometry monitoring of CD1a, CD14, and CD11c markers (Supplementary Figure 1).

### Peripheral Blood Lymphocytes Activation

Peripheral blood lymphocytes (PBL) were cultivated at 37°C for 4 days with or without different stimuli: 1:1 αCD3/CD28 coated Dynabeads (Invitrogen) [coated according to manufacturer protocol with 4 μg anti-CD3, clone OKT3 (Biolegend) and 2 μg anti-CD28, clone CD28.2 (BD Pharmingen)], 100 ng/ml IL-15 (Miltenyi), or 1 μg/ml crosslinked CD40L and 20 ng/ml TNFα (Miltenyi). Cells were cultivated in either XVivo-15 serum-free medium (Lonza) for PBL stimulated with αCD3/CD28 beads or in complete RPMI for PBL treated with IL-15 or CD40L+TNFα.

### Flow Cytometry

Phenotyping was performed with antibodies presented in Supplementary Table 1. For macrophages staining experiment, cells were recovered from culture plates by gentle scrapping. TAM-R phenotyping was performed using the following Phycoerythrin (PE) conjugated antibodies: mouse IgG1 α-MerTK (Biolegend, clone 590H11G1E3), mouse IgG1 α-Tyro3 (R&D Systems, clone 96201), mouse IgG1 α-Axl (R&D Systems, clone 108724), or their respective isotypic controls. Cells were analyzed using MACSQuant Analyzer X (Miltenyi Biotech) and FlowJo software (BD). Gating was performed on single cells and live cells before applying the strategy depicted in Supplementary Figure 2A.

### Gas6/PROS1 Production Quantification

Supernatant from moDC and macrophages differentiation were harvested at the end of differentiation. CD4^+^ T cells were negatively sorted from Peripheral blood mononuclear cells (PBMC) via magnetic separation (Miltenyi) and seeded at 0.5 · 10^6^ cells/ml in serum-free XVivo-15 medium (Lonza) with 1:1 αCD3/CD28 coated beads for 4 days before supernatant harvest. Samples were stored at −80°C before further analysis. Gas6 and PROS1 concentrations were measured using a commercially available multiplex magnetic beads-based kit (Luminex, R&D Systems) following the manufacturer's protocol. All samples and standards were assayed in duplicate. Plates were read on a Magpix instrument (Luminex), and data were analyzed using xPONENT software (Luminex) using a five-parameter fit to calculate concentrations.

### Human Recombinant PROS1 Production

Full-length PROS1 cDNA was synthetized with addition of a 6xHis Tag in C-Term and cloned into pcDNA™3.4 vector (Life Technologies) by GeneArt (ThermoFisher Scientific). Plasmid was transfected into Expi293 expression systems (Gibco) according to manufacturer's instructions. Expression of PROS1 was performed in presence of 10 μg/ml vitamin K (Sigma). Three days post-transfection, the supernatant was harvested, and PROS1 was purified on HisTrap column (GE Healthcare) using a linear gradient of 20–500 mM imidazole in 20 mM Tris and 500 mM NaCl, with the addition of 2 mM CaCl_2_ in all buffers. PROS1-containing fractions were pooled and dialyzed overnight against phosphate-buffered saline (PBS) containing 2 mM CaCl_2_.

### moDC Cytokines Production Assay

Differentiated moDC and moDC pretreated 24 h with Dex were transferred to XVivo-15 medium. Cells were seeded at 1 · 10^6^ cells/ml and treated with 100 ng/ml LPS (Invivogen), 10 μg/ml PROS1, and/or 1 μM UNC2025 (Selleckchem) or dimethylsulfoxide (DMSO) (vehicle). Cells were incubated 48 h at 37°C 5% CO_2_. Supernatants were collected and conserved at −80°C. Cells viability was assayed in cytometry using Zombie viability dye (Biolegend). IL-10 was measured in the supernatant using an ELISA kit (BD Bioscience) according to manufacturer's recommendations. Plates were read at 450 nm with correction at 570 nm using a Spark microplate reader (TECAN).

### Immunohistochemistry

Multi-organ tumor and healthy formalin-fixed paraffin-embedded (FFPE) tissue microarrays (TMA) were obtained from US Biomax, Inc. (USA), and stained with anti-MerTK antibody (ab52968, Abcam). Staining was revealed with horseradish peroxidase (HRP) coupled secondary antibody and diaminobenzidine from EnVision Detection Systems (Agilent), then counterstained with hematoxylin. Immunohistochemistry (IHC) interpretation and H-score reporting were performed by a pathologist. H-score was calculated by multiplying an intensity score (0–4) with a distribution score (0–4) for a final range of 0–16.

### Data Analysis

Results are represented as one point per *n*, and the median is represented with a horizontal bar. The number of *n* corresponds to the number of independent experiments using cells obtained from different donors. Statistical difference was determined using Wilcoxon rank-sum test, paired *t*-test, or Friedman's test as indicated. Statistical significance was defined as *p* < 0.05. Analysis and graphing were conducted using Prism Software (GraphPad).

## Results

### MerTK Is a Marker of Immunosuppressive Myeloid Cells

In order to simultaneously analyze the expression of the three TAM-R, we generated dendritic cells (DC) *in vitro* from monocytes using granulocyte-macrophage colony-stimulating factor (GM-CSF) and IL-4, various *in vitro* differentiated macrophages, i.e., M1, M2a, and M2c as described by Martinez et al. (23), and tumor-associated-like macrophages (TCM), generated with conditioned supernatant from triple-negative breast cancer cell line MDA-MB-231 (24).

**Figure 1:**
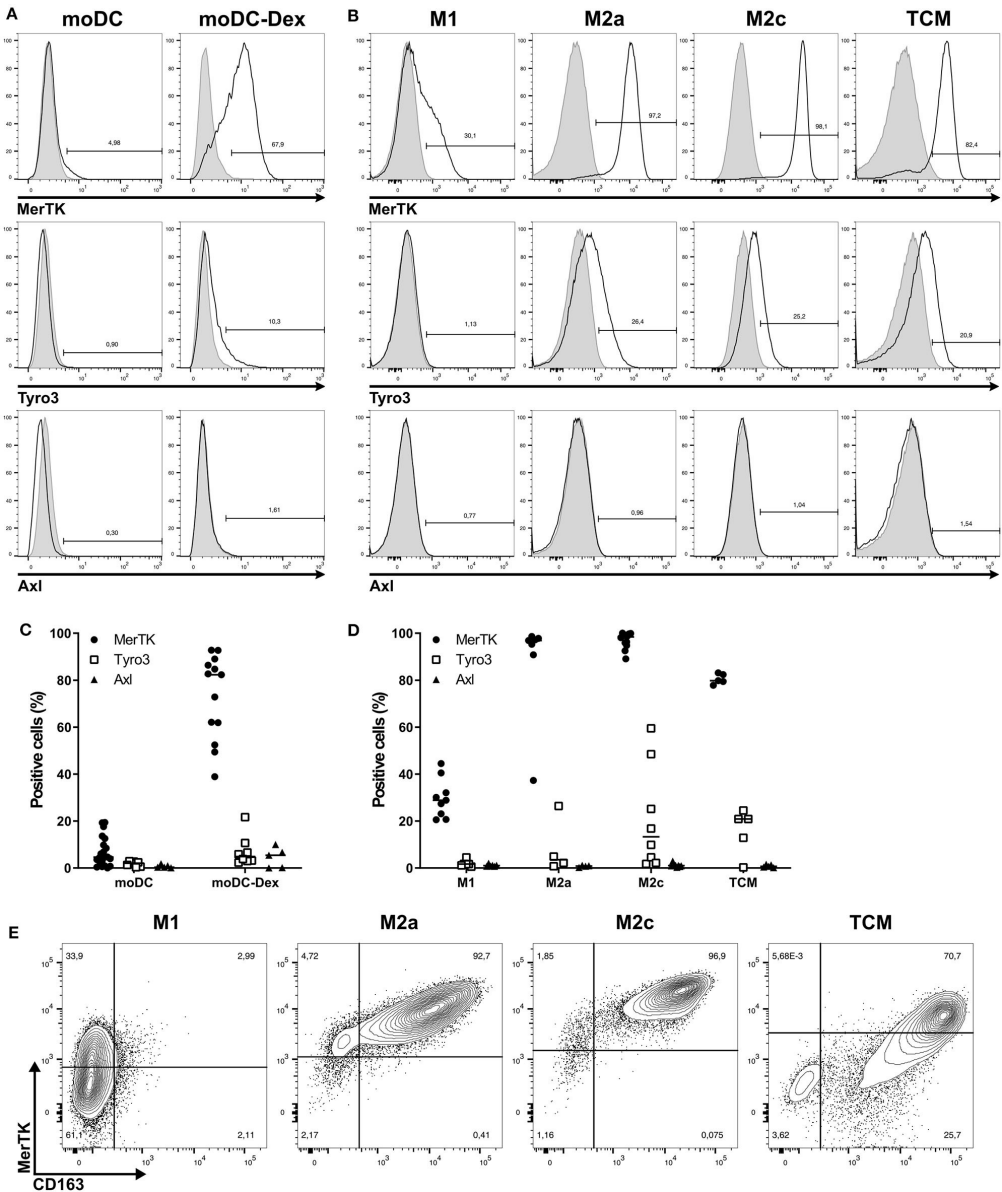
Among TAM-R, MerTK is expressed by suppressive myeloid cells. TAM-R receptors expression was assessed by flow cytometry (*n* > 5) on moDC in the presence or not of dexamethasone and *in vitro* polarized M1, M2a, and M2c macrophages, and tumor conditioned macrophages (TCM) (*n* = 4–5). **(A,B)** Representative histograms of MerTK, Tyro3, and Axl expression for one donor of moDC treated (moDC-Dex) or not (moDC) by dexamethasone **(A)** or M1, M2a, M2c, and TCM macrophages **(B)**. Positive staining is represented by a black line, and isotypic controls by a gray filled line. **(C,D)** Summary of all donors for moDC **(C)** and macrophages **(D)**. MerTK expression is represented by full dots, Tyro3 by hollow square, and Axl by full triangles. **(E)** MerTK and CD163 expression in a representative donor for M1, M2a, M2c, and TCM. Quadrant gates were placed based on isotypic controls staining.

As shown in Figures 1A,C, MerTK was the only TAM-R slightly expressed on immature moDC, with a median of 4.65%. Induction of a tolerogenic phenotype on moDC with dexamethasone (Dex) as described by Cabezón et al. (5) induced MerTK expression on 80% of cells. While Dex is known to regulate MerTK expression, it did not induce Tyro3 and Axl (Figure 1A). In a similar manner, MerTK is highly expressed (>90%) by immunosuppressive macrophages M2a (M-CSF + IL-4) and M2c (M-CSF + IL-10 + TGFβ), while M1 (GM-CSF+IFNγ) macrophages supporting proinflammatory functions weakly express MerTK (median: 28.9%, Figure 1B). Expression of Tyro3 is very heterogeneous on M2c macrophages with a median expression of 13.35% including some donors with up to 60% of Tyro3 expression (Figure 1D). Tumor-conditioned macrophages were obtained by differentiation of monocytes during 7 days with 50% of supernatant from a triple-negative breast cancer cell line, MDA-MB-231, which induces a strong M2 phenotype (24). TCM displayed a strong expression of MerTK (79.8%) as well as some expression (20.9%) of Tyro3. Furthermore, MerTK expression in macrophages correlated with expression of the M2 marker CD163 (Figure 1E). In M2a, M2c, and TCM, MerTK expression was higher in CD163^high^ cells, whereas MerTK expression was low in CD163^neg^ M1 macrophages. Taken together, these results indicate that MerTK is a marker of immunosuppressive myeloid cells such as M2 macrophages.

### MerTK Expression in Stromal Mononuclear Cells

In order to validate the presence of MerTK in immune infiltrate, we performed an IHC study on a commercially available FFPE-multiple organ tumor TMA (US Biomax, Inc.) which contained 208 tumor cores, each from a different donor, including 20 tumor tissues (Supplementary Table 2). One core was of insufficient quality to be interpreted. A supplementary TMA of 32 healthy tissues each from three normal human individuals was stained in parallel as control.

**Figure 2:**
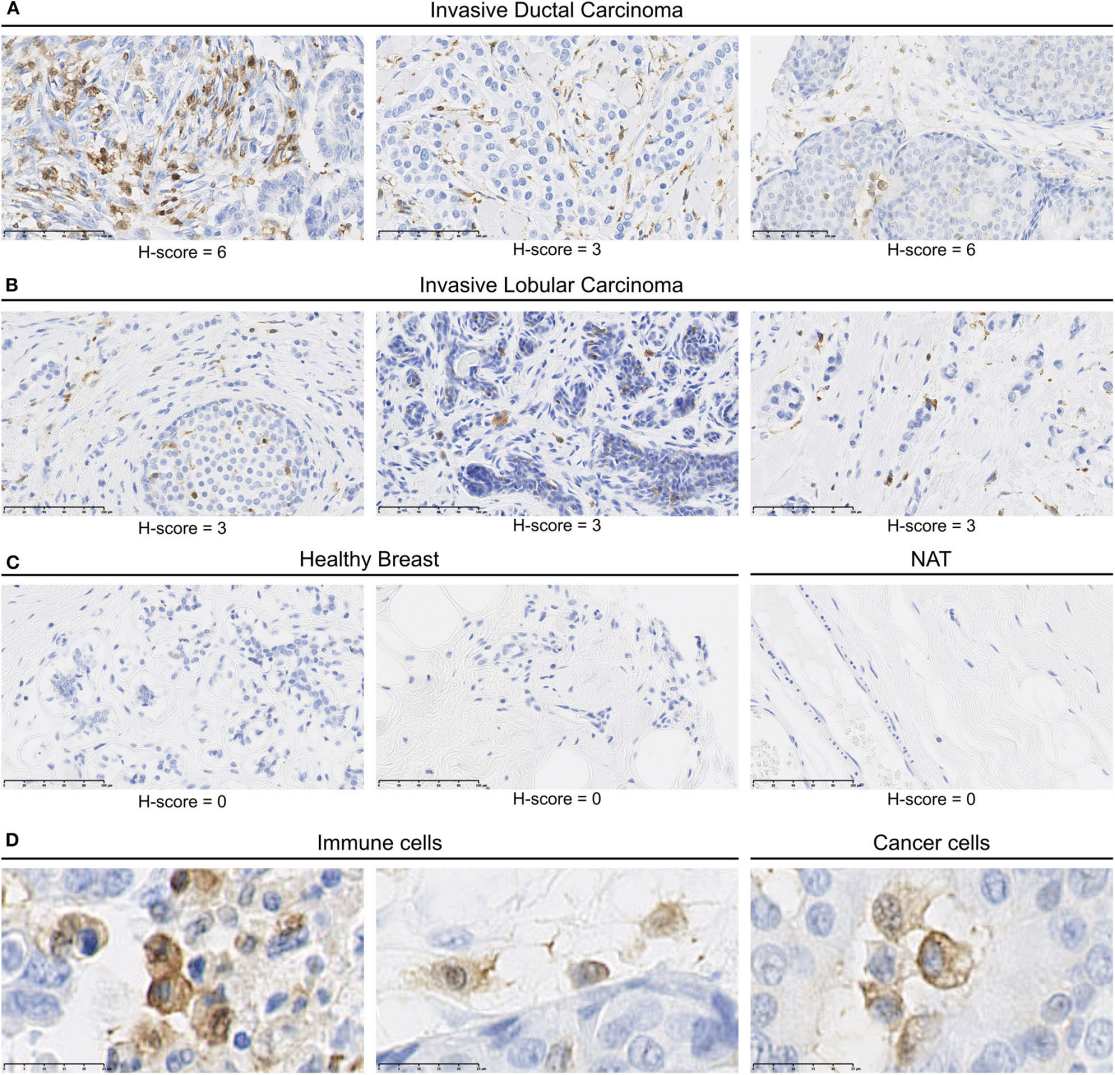
MerTK is expressed by mononuclear cells in the immune infiltrates of human breast cancer tumors. Immunohistochemical staining of a multiple organ tumor and healthy tissue arrays from US Biomax, Inc. **(A)** MerTK staining in three invasive ductal carcinoma patients. **(B)** MerTK staining in three invasive lobular breast carcinoma patients. **(C)** MerTK staining in healthy breast tissue (left) or in normal tissue adjacent to the tumor (NAT, right). **(D)** Selected enlargement of immune cells (left) and cancer cells (right) in invasive ductal carcinoma patients. Bottom left scale bar represents 100 μm in **(A**–**C)** and 25 μM in **(D)**. Counterstained with hematoxylin.

We identified MerTK expression in multiple cancer tissues, expressed either by cancers cells or by mononuclear cells in the stroma (Supplementary Table 2). In summary, a total of 119/207 core presenting positive MerTK staining in either stromal mononuclear cells (71/207), cancer cells (34/207), or both (14/207) were observed in the tumor TMA. For example, three out of four glioblastoma samples displayed a positive MerTK staining on cancer cells. On the other hand, among breast cancer, we found seven out of eight invasive ductal carcinomas (Figure 2A) and four out of eight invasive lobular carcinomas (Figure 2B) presenting a positive staining for MerTK in immune infiltrate. In one out of eight invasive ductal carcinomas, both immune infiltrate and cancer cells positive for MerTK were observed (Figure 2A, left and Figure 2D, middle and left). H-score for positive staining comprised between 3 and 6 on a 0–16 scale. This relatively low H-score is the result of a low distribution score, which can be explained by an expected low proportion of immune infiltrating cells compared to cancer cells. MerTK expression was absent in breasts from healthy patients or in normal tissue adjacent to tumor (Figure 2C).

MerTK expression was also observed in immune infiltrate in other tumor types such as in pancreas duct adenocarcinoma, in head and neck, skin and cervix squamous cell carcinoma, in ovary, uterus, and prostate adenocarcinoma as well as in kidney clear cell and granular cell tumors (Supplementary Table 2). Moreover, MerTK expression was more frequently observed in immune infiltrate than on cancer cells but rarely on both. Taken together, these results demonstrate the presence of MerTK in immune infiltrate and on tumor cells in multiple solid cancers.

### Myeloid Cells Secrete TAM-R Ligands Gas6 and PROS1

**Figure 3:**
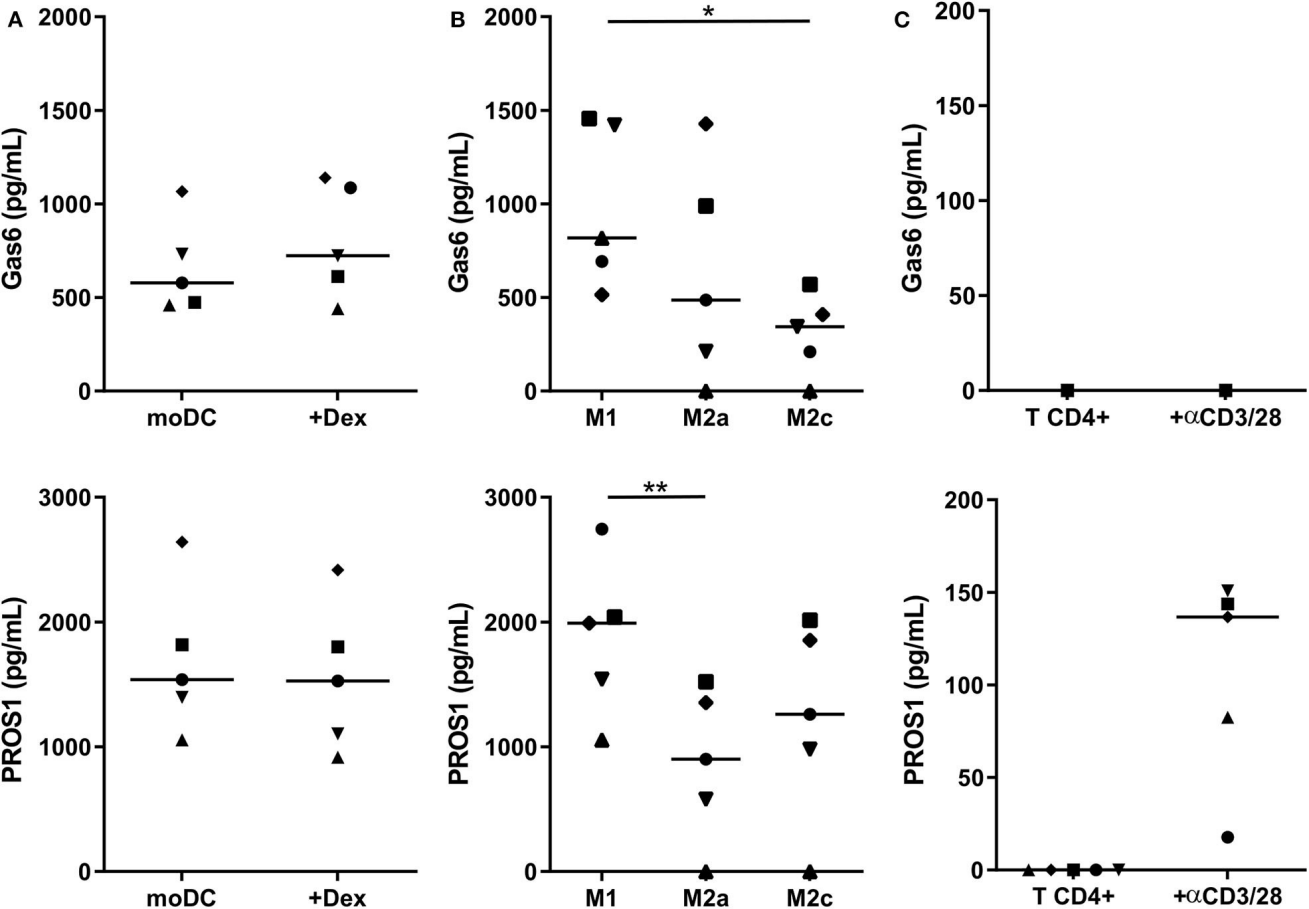
Gas6 and PROS1 secretion by *in vitro* differentiated myeloid cells. Gas6 and PROS1 concentration was measured by a multiplex bead assay **(A)** in supernatants from *in vitro* differentiated DC treated or not by dexamethasone (Dex) (*n* = 5) **(B)** in supernatants from *in vitro* derived macrophages (*n* = 5) **(C)** in supernatants from purified T CD4^+^ cells with or without 4 days stimulation with anti CD3/CD28 antibodies (*n* = 5). Statistical significance was determined using Friedman's test (*p < 0.05; **p < 0.01).

We then investigated whether human myeloid cells also produce their own TAM-R ligands. Using a multiplexed immunodetection assay, we observed that moDC and immunosuppressive moDC-Dex secrete, respectively, a median of 577.4 and 723.7 pg/ml of Gas6 and 1,538.2 and 1,527.4 pg/ml of PROS1 without activation (Figure 3A). Thus, moDC and moDC-Dex produce equal quantities of TAM-R ligands. Both M1 and M2 macrophages produce Gas6 and PROS1 in resting state (Figure 3B). Interestingly, M1 secrete significantly more PROS1 than M2a macrophages (1,991.6 and 901.5 pg/ml, respectively, *p* < 0.01). M1 macrophages also produce more Gas6 than M2c macrophages (818 and 343.9 pg/ml, respectively, *p* < 0.05). M2a and M2c macrophages produced an equivalent amount of Gas6 and PROS1. Following a report by Carrera Silva et al. (25) of PROS1 at the surface of T cells, we also investigated whether CD4^+^ T cells produced Gas6 and PROS1 after T cell receptor (TCR) activation (stimulation by the anti-CD3/CD28 antibody pair). Gas6 was not detected in CD4^+^ culture supernatant, whereas PROS1 level was 136.6 pg/ml following activation (Figure 3C).

Taken together, these results indicate that MerTK expression is not correlated with a higher TAM-R ligands production in myeloid cells. Gas6 and Pros1 are ubiquitously produced by *in vitro* derived myeloid cells, suggesting that they could be a source of ligands within the tumor microenvironment.

### PROS1 Increases Immunosuppressive Cytokines Secretion in Tolerogenic moDC

We next evaluated if Gas6 and PROS1 ligands may influence myeloid cell functions through an autocrine effect. As we showed that MerTK is primarily expressed by human immunosuppressive myeloid cells, we hypothesized that MerTK activation could contribute to their immunosuppressive functions. IL-10 is an essential cytokine implicated in immunosuppression, notably produced by immunosuppressive myeloid cells. Thus, we investigated the effect of PROS1 in moDC and tolerogenic moDC after LPS activation on the production of IL-10.

**Figure 4:**
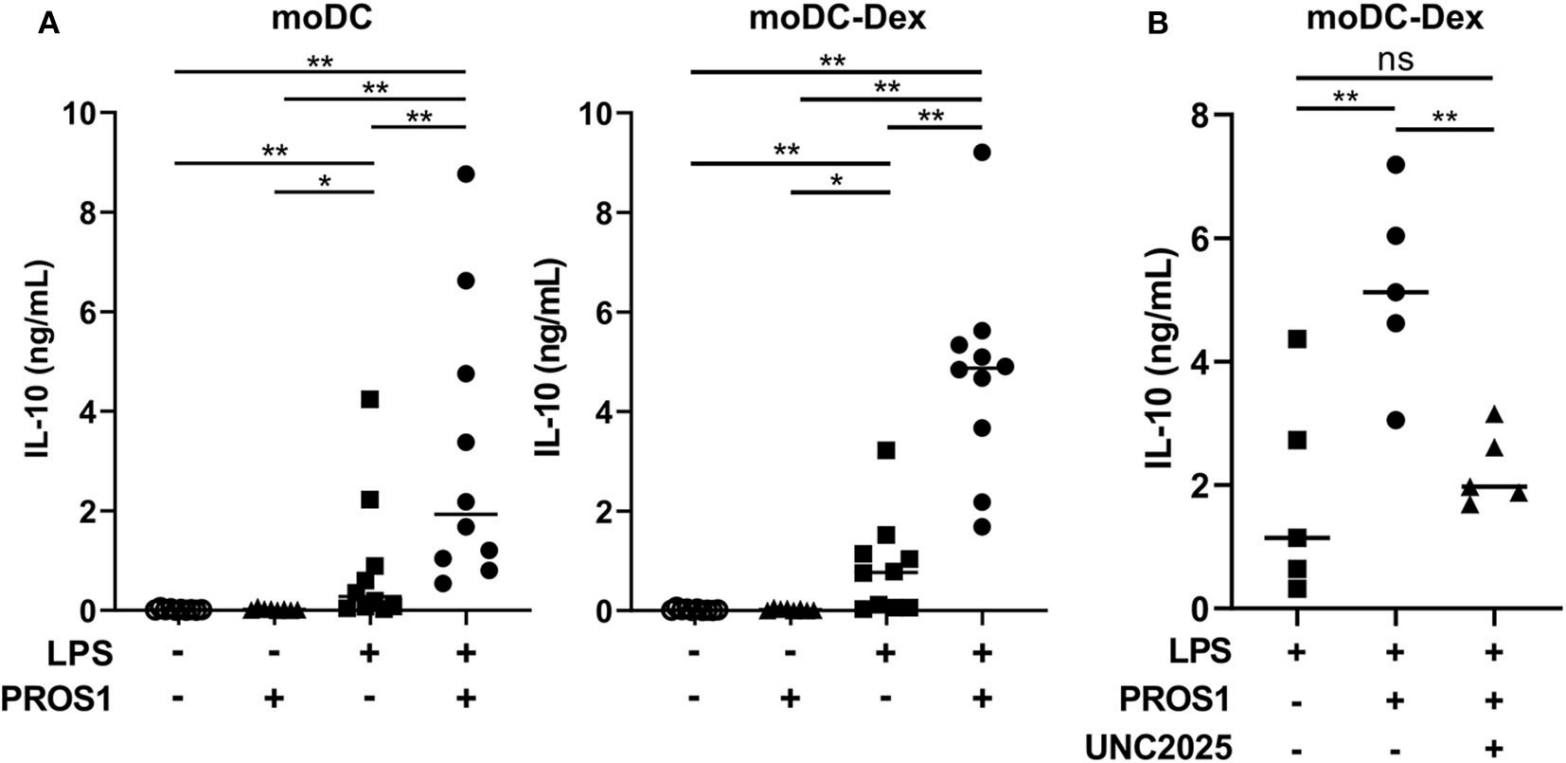
PROS1 enhances LPS-inducible IL-10 secretion in MerTK^high^ moDC. **(A)** IL-10 was quantified by ELISA from supernatants of moDC or moDC-Dex stimulated by LPS (100 ng/ml) and/or PROS1 (10 μg/ml) (*n* = 10). Statistical significance was determined with a Wilcoxon rank-sum test. **(B)** IL-10 was quantified by ELISA from supernatants of moDC-Dex incubated during 48 h with LPS, PROS1, and/or UNC2025 (1 μM) (*n* = 5). Statistical significance was determined with paired *t*-test (**p < 0.01; *p < 0.05; ns, not significant).

For this purpose, we generated a γ-carboxylated recombinant human PROS1, based on the work of Tsou et al. (7) that demonstrated the necessity to add vitamin K in the expression medium to obtain a functional protein. We then stimulated moDC and moDC-Dex with LPS (100 ng/ml) in the presence or not of recombinant PROS1 (10 μg/ml) (Figure 4A). In the absence of LPS and with or without PROS1, neither moDC nor moDC-Dex produced IL-10. As expected, after LPS stimulation, IL-10 production was strongly increased to a median of 768.8 pg/ml in MerTK^high^ moDC-Dex. Addition of PROS1 to LPS stimulated moDC-Dex and further increased IL-10 production by 6.3-fold to 4,872.7 pg/ml. To a lesser extent, IL-10 production was elevated from 274.4 to 1,927.6 pg/ml after PROS1 addition in LPS-treated MerTK^low^ moDC (Figure 4A). MerTK^high^ moDC-Dex produced 2.5 times more IL-10 in response to LPS and PROS1 than MerTK^low^ moDC. Furthermore, inhibition of MerTK activity by the small molecule inhibitor UNC2025 (26) blocked PROS1-mediated IL-10 production in moDC-Dex, suggesting a direct effect of PROS1/MerTK signaling (Figure 4B).

### MerTK Is Expressed by Activated T and B Lymphocytes

Besides myeloid cells, the tumor microenvironment is composed of several other immune cell populations that may either participate in tumor immunity (e.g., NK cells/cytotoxic T CD8^+^ cells) or sustain an immunosuppressive environment [e.g., regulatory T cells (T_regs_)], but to date, MerTK, Tyro3, and Axl expression in human lymphoid cells are poorly characterized.

**Figure 5:**
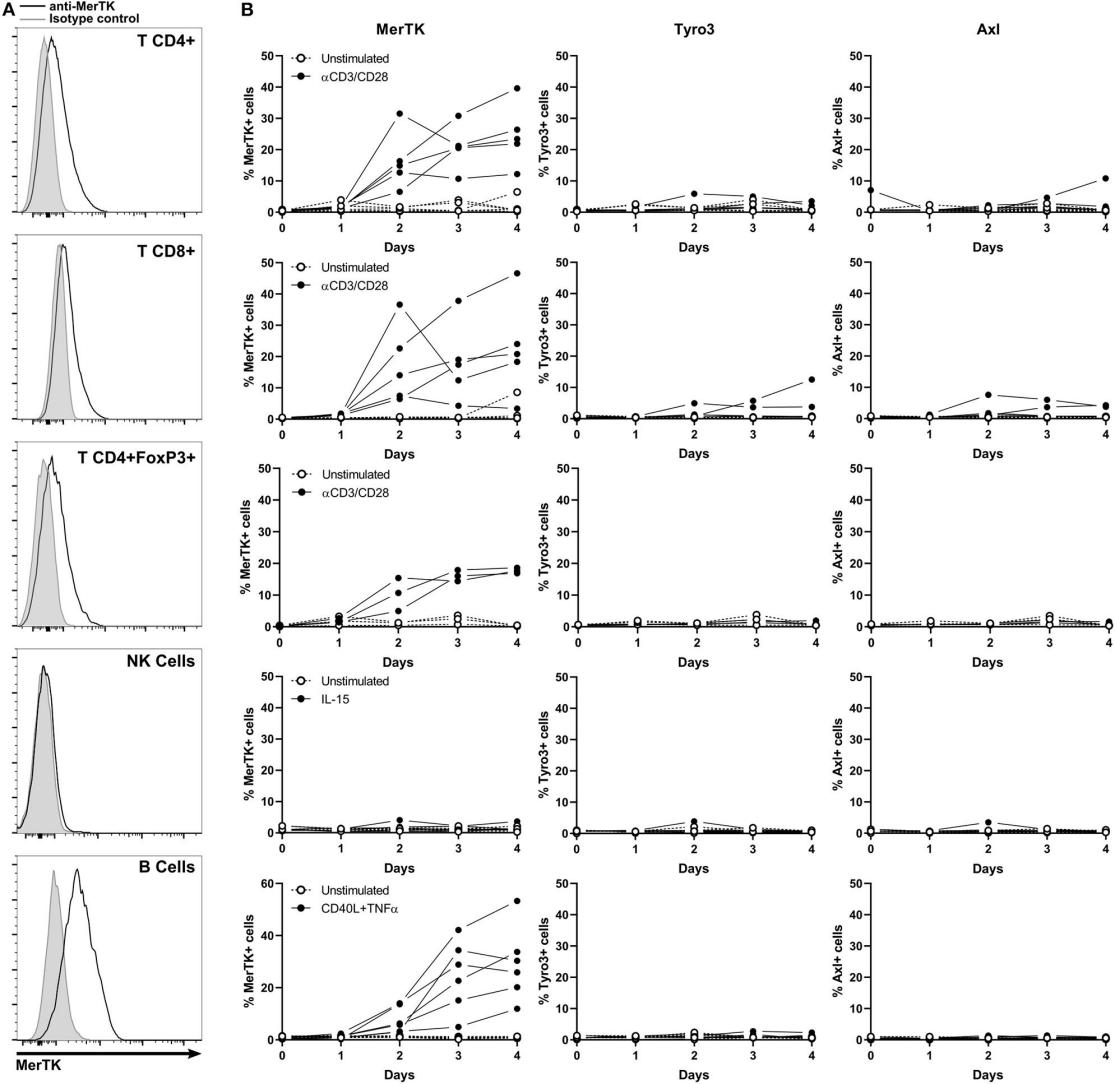
MerTK is expressed on activated T and B cells. **(A)** Flow cytometry analysis of MerTK expression in activated CD4^+^, T CD8^+^, CD4^+^FoxP3^+^ T lymphocytes, and NK and B cells from PBL. A representative donor is shown. Gray histogram corresponds to isotype control, and MerTK staining is represented by a black line. **(B)** Kinetic of cell surface TAM-R expression performed on PBL and analyzed by flow cytometry. Full dots represent activated samples, and hollow dots represent unstimulated samples, with one dot per donor. T CD4^+^ and CD8^+^, *n* = 5; T CD4^+^FoxP3^+^, *n* = 3; NK and B cells, *n* = 6.

Thus, we investigated TAM-R expression in T, NK, and B cells, both in resting and activated states. For this purpose, we isolated peripheral blood lymphocytes (PBL) that we stimulated with anti-CD3/CD28 coated beads, IL-15, or a mix of crosslinked CD40L and TNFα for T cells, NK, and B cells, respectively. We then measured MerTK, Tyro3, and Axl expression by flow cytometry every 24 h for 4 days (Figure 5). Activation status was followed on T and NK cells by monitoring PD1 and CD69 expression, respectively (Supplementary Figure 2B).

MerTK started to be expressed on the surface of CD4^+^, CD8^+^, and CD4^+^FoxP3^+^ T cells after 2 days of anti-CD3/CD28 activation, until it reached about 20% expression at day 4. Tyro3 and Axl remained undetectable on resting and stimulated T cells. TAM-R expression in activated human B cells remained unexplored until now. Surprisingly, up to 50% of B cells expressed MerTK when stimulated 4 days with a cocktail of CD40L and TNFα, but not at a resting state. Neither Tyro3 nor Axl were detected in B cells. We could not detect surface expression of any TAM-R in resting or IL-15 activated NK.

MerTK expression on activated B and T lymphocytes suggests that the presence of TAM-R ligands in tumor microenvironment which are produced by myeloid cells might have a functional paracrine effect on infiltrating lymphoid cells.

## Discussion

We analyzed the expression profile of the TAM-R on human immune cells. To our knowledge, this is the first study which compared exhaustively the expression of the three TAM-R, Tyro3, Axl, and MerTK, in both human myeloid and lymphoid cells with appropriate stimuli and revealed the unique expression profile of MerTK among its family in human immune cells. As heterodimerization or cross-phosphorylation have been observed within the TAM-R family (16, 27), evaluating all three receptors together is essential.

We generated myeloid cells *in vitro* using well-known stimuli to obtain immunosuppressive macrophages and tolerogenic DC. MerTK expression in macrophages is associated with the immunosuppressive M2 phenotype. In moDC, dexamethasone is the most described tolerogenic stimulus to induce MerTK expression (15). However, the expression of TAM-R in *in situ* immunosuppressive macrophages or tolerogenic DC remains unknown. Our results encourage further studies to explore TAM-R expression in immunosuppressive macrophages and tolerogenic DC isolated from fresh tumors.

MerTK is an essential receptor for efferocytosis (10, 28), a process that has been demonstrated to promote an immunosuppressive environment allowing tumor growth and metastasis in *in vivo* models of breast cancer (18, 19, 29). We demonstrated that MerTK is associated with a M2 phenotype in macrophages, which are associated with a worsened clinical prognostic (30). These results suggest that within an immunosuppressive cancer microenvironment, MerTK is expressed at the surface of myeloid cells and could participate in efferocytosis-mediated immunosuppression. Furthermore, in MerTK^−/−^ mice tumor models, efferocytosis is impaired leading to the decrease of immunosuppressive cytokines associated with tumor growth (19).

Furthermore, we observed MerTK expression *in situ* in multiple solid tumor immune infiltrates such as in breast, ovary, uterus, and cervix cancer, as well as in prostate, skin, head, and neck cancer. Those cells display a myeloid morphology for most of them and are found in a wide variety of solid tumors. Taken together, these results suggest that MerTK^+^ immunosuppressive myeloid cells are common within the tumor microenvironment and as such represent potential targets for therapies. Additionally, the presence of MerTK was observed in some cases in a lower number of smaller and round mononuclear cells, morphologically consistent with large lymphocytes, potentially B cells. Further characterization of MerTK^+^ immune infiltrating cells to determine their lineages and phenotypes will be required. Although using a TMA does not allow us to perform quantitative or clinical evaluation of MerTK *in situ*, it provides an interesting overview of MerTK frequent expression in various cancers, supporting its potential as a target for cancer therapy.

We have demonstrated that both ligands Gas6 and PROS1 are ubiquitously produced by myeloid cells. Preliminary results did not show any striking effect of LPS activation on myeloid cell-mediated ligand production (data not shown). This observation is corroborated by the work of Scutera et al. (31) showing that LPS and Poly:IC did not regulate Gas6 mRNA level in moDC. Taken together, these results suggest that myeloid cells represent a constitutive source of Gas6 and PROS1 in the tumor microenvironment. Cancer cells are also known to produce variable levels of ligands (16).

TAM-R expression has been described in multiple cancer indications, with cancer-promoting roles such as tumor growth, survival, chemoresistance, or metastasis promotion [reviewed in (16)]. It remains, however, unclear how tumor and myeloid cells crosstalk could occur through ligands/TAM-R signaling. Recently, it was demonstrated that tumor cells-secreted PROS1 could inhibit M1 polarization in mouse models and thus reduced anti-tumor immune response (21). These results suggest that accumulation of ligands in the microenvironment produced either by myeloid cells or by cancer cells participate in both immunosuppression and cancer cell growth. Thus, a MerTK targeting strategy could present a dual effect on both reversing immunosuppression and slowing down cancer growth.

Although MerTK implication in immunosuppression is known, its mechanism remains unclear. It had been reported previously that MerTK- or PROS1-deficient mice display lower levels of IL-10 production by myeloid cells following bacterial or viral activation, or in cancer models (18, 32, 33). We present evidence that MerTK activation by its ligand PROS1 induces IL-10 production in human tolerogenic DC following TLR4 activation. A similar regulation of IL-10 production has also been demonstrated in human M2 macrophages following Gas6 stimulation (10) suggesting a redundant effect of Gas6 and PROS1 on MerTK^+^ myeloid cells.

In the context of cancer, cell death induced by cytotoxic chemotherapeutic treatment releases cell death-associated molecular patterns that are capable of activating TLR pathways. One of such factors, high mobility group box protein 1 (HMGB1), has been shown to bind to TLR4 (34). TAM-R signaling has been shown to inhibit TLR response via the production of SOCS1/3 (13, 35). In addition to our data, these results suggest that MerTK could hinder immunogenic cancer cell death response by inhibiting TLR pathways directly, and indirectly by inducing production of immunosuppressive cytokines such as IL-10. Thus, a combination of cytotoxic treatment with MerTK targeting agent may shift the balance toward immunogenic cell death. As a matter of fact, two recent studies in mouse models of breast and lung cancer demonstrated that blockade of efferocytosis with an anti-MerTK antibody promotes tumor antigen presentation by infiltrating myeloid cells and triggers Type I IFN immune response as well as CD8^+^ T cells and NK mediated tumor immunity, resulting in tumor rejection and abscopal effect (36, 37).

We observed MerTK but not Tyro3 or Axl expression in TCR-activated human T lymphocytes. Although previous reports of MerTK in T CD4^+^ and CD8^+^ have been published (15, 22), we discovered that FoxP3^+^CD4^+^ T_regs_ also express MerTK upon activation. It was also demonstrated that activated CD4^+^ and CD8^+^ T lymphocytes express higher cell surface PROS1 than resting cells (22, 25). As PROS1 is a secreted factor, we expected it to be found in large quantities on activated CD4^+^ T cell supernatants. However, we found little PROS1 in those supernatants. PROS1 was demonstrated to be an essential co-factor of T cell proliferation through MerTK (15, 22), indicating that T cells may have consumed most of the PROS1 they produced in an autocrine amplification loop. Furthermore, MerTK expression on T cells which was upregulated tardily could support its potential control of the magnitude and the duration of T effector function.

We hereby report for the first time that CD40L+TNFα-activated human B cells express MerTK, while it is absent in resting state. Previous studies showed the absence of MerTK in resting mouse B cell lineage (38, 39), while another reported the presence of MerTK in mouse B cells in a model of chronic graft-vs.-host disease (40). In those models, mice develop a chronic inflammatory environment where B cells are constantly activated and produce autoantibodies, consistent with CD40L and TNFα stimulation. MerTK function in human B cells remains, however, unknown. In a similar mechanism to what has been described in T cells, MerTK could be a costimulatory receptor that increases B cell activation in the presence of PROS1 (15, 22). MerTK expression in human B cells opens exciting research perspectives to explore TAM-R biology in this lymphoid cell subset and its components, such as regulatory B cells. Interestingly, CD40L activation induces IL-10 production in a subset of B cells (41). MerTK upregulation following CD40L activation may be caused by or responsible for this IL-10 production.

We did not observe MerTK expression in NK cells, whether activated or resting. MerTK expression had previously been observed in murine splenic NK, but not in human circulating NK (38, 42, 43). Our observations are consistent with the previous literature; however, considering data from mouse models, we cannot rule out that certain stimuli may trigger MerTK expression in human NK cells.

In conclusion, we provide an in-depth characterization of Tyro3, Axl, and MerTK expression in human immune cells demonstrating that MerTK is the most prominent TAM-R expressed on immunosuppressive myeloid cells. Our data expand current understanding of MerTK regulation of DC activation, demonstrating that MerTK upregulation results in higher IL-10 production through its activation by PROS1. Developing therapies which selectively target MerTK among TAM-R could be a promising approach to alleviate myeloid-mediated immunosuppression in cancer.

## Data Availability Statement

The raw data supporting the conclusions of this article will be made available by the authors, without undue reservation.

## Ethics Statement

Ethical review and approval was not required for the study on human participants in accordance with the local legislation and institutional requirements. The Ethics Committee waived the requirement of written informed consent for participation.

## Author Contributions

PG, VD, and JV-G designed the research and wrote the manuscript. PG, SR, LG, and AC performed experiments, analyzed, and visualized the data. JM and CC supervised the project. TM and CR revised the manuscript. All authors contributed to the article and approved the submitted version.

## Conflict of Interest

PG, SR, LG, AC, TM, CR, JM, and VD were employed by Elsalys Biotech. The funder had the following involvement with the study: Elsalys Biotech assesses MerTK as a potential therapeutic target. PG was the recipient of a fellowship from the Association Nationale de la Recherche et de la Technologie (ANRT n°2016/1465). The remaining authors declare that the research was conducted in the absence of any commercial or financial relationships that could be construed as a potential conflict of interest.
