# Supplementary material for: Expression of TAM-R in Human Immune Cells and Unique Regulatory Function of MerTK in IL-10 Production by Tolerogenic DC
Source: Front Immunol. 2020 Sep 25;11:564133. doi: 10.3389/fimmu.2020.564133 (PMC7546251; doi:10.3389/fimmu.2020.564133)
Supplement: Supplementary file 1 [file Table_1.DOCX]

Supplemental Table I: Antibodies used for T, NK, and B cells phenotyping

| **Marker** | **Fluorochrome** | **Clone** | **Manufacturer** |
| --- | --- | --- | --- |
| *A : Panel for T cells phenotyping* | | | |
| CD3 | PerCp-Cy5.5 | OKT3 | Biolegend |
| CD4 | PE-Cy7 | SK3 | BD |
| CD8 | BV510 | RPA-T8 | BD |
| PD1 | APC | MIH4 | BD |
| CD56 | Viobright 515 | REA196 | Miltenyi |
| Viability | Zombie NIR | *n/a* | Biolegend |
| *B : Panel for NK cells phenotyping* | | | |
| CD56 | Viobright 515 | REA196 | Miltenyi |
| CD69 | PE-Cy7 | FN50 | BD |
| CD3 | PerCp-Cy5.5 | OKT3 | Biolegend |
| CD20 | PerCp-Cy5.5 | 2H7 | Biolegend |
| Viability | Zombie Violet | *n/a* | Biolegend |
| *C : Panel for B cells phenotyping* | | | |
| CD20 | BV510 | 2H7 | Biolegend |
| CD3 | PerCp-Cy5.5 | OKT3 | Biolegend |
| CD14 | PerCp-Cy5.5 | MφP9 | BD |
| CD56 | PerCp-Cy5.5 | B159 | BD |
| CD11c | PerCp-Cy5.5 | B-ly6 | BD |
| Viability | Zombie Violet | *n/a* | Biolegend |
