# Supplementary material for: Expression of TAM-R in Human Immune Cells and Unique Regulatory Function of MerTK in IL-10 Production by Tolerogenic DC
Source: Front Immunol. 2020 Sep 25;11:564133. doi: 10.3389/fimmu.2020.564133 (PMC7546251; doi:10.3389/fimmu.2020.564133)
Supplement: Supplementary file 2 [file Table_2.DOCX]

**Supplemental Table II:** **MerTK is found both on cancer cells and in immune infiltrate as revealed by IHC study on a multi-tumor TMA.** Numbers indicate number of positive or negative cores/total number of cores in indication

| **Organ** | **Diagnosis** | **Negative Cores** | **Cores with positive cancer cells** | **Core with positive mononuclear cells** |
| --- | --- | --- | --- | --- |
| **Stomach** | Adenocarcinoma | 4/7 | 2/7 | 1/7 |
| **Esophagus** | Adenocarcinoma | 2/4 | 1/4 | 1/4 |
|  | Squamous Cell Carcinoma | 2/4 | 1/4 | 1/4 |
| **Colon** | Adenocarcinoma | 4/8 | 2/8 | 3/8 |
| **Liver** | Hepatocellular carcinoma | 8/8 | 0/8 | 0/8 |
| **Pancreas** | Duct adenocarcinoma | 2/8 | 1/8 | 5/8 |
| **Lungs** | Squamous cells carcinoma | 3/4 | 0/1 | 1/4 |
|  | Adenocarcinoma | 2/4 | 1/4 | 1/4 |
|  | Large cell carcinoma | 2/4 | 2/4 | 1/4 |
|  | Small cell carcinoma | 4/4 | 0/4 | 0/4 |
| **Cerebrum** | Astrocytoma | 1/4 | 3/4 | 0/4 |
|  | Glioblastoma | 1/4 | 3/4 | 0/4 |
| **Spleen** | B-Cell Lymphoma | 2/8 | 6/8 | 0/8 |
| **Lymph Node** | Hodgkin Lymphoma | 0/4 | 4/4 | 0/4 |
|  | Follicular, T Cell and Large Cell lymphoma | 3/4 | 1/4 | 0/4 |
| **Thyroid** | Papillary Carcinoma | 1/4 | 3/4 | 1/4 |
|  | Follicular Carcinoma | 3/4 | 1/4 | 1/4 |
| **Head & Neck** | Squamous Cell Carcinoma | 2/8 | 2/8 | 6/8 |
| **Skin** | Squamous Cell Carcinoma | 2/8 | 0/8 | 6/8 |
|  | Dermatofibrosarcoma | 3/4 | 0/4 | 1/4 |
|  | Mixoid liposarcoma | 3/4 | 1/4 | 0/4 |
|  | Malignant melanoma | 6/8 | 1/8 | 1/8 |
| **Breast** | Invasive ductal carcinoma | 1/8 | 1/8 | 7/8 |
|  | Invasive lobular carcinoma | 4/8 | 1/8 | 4/8 |
| **Ovary** | Adenocarcinoma | 0/8 | 2/8 | 8/8 |
|  | Disgerminoma | 4/4 | 0/4 | 0/4 |
|  | Sertoli-cell tumor | 2/4 | 0/4 | 2/4 |
| **Uterus** | Adenocarcinoma | 1/8 | 0/8 | 7/8 |
| **Cervix** | Squamous cell carcinoma | 0/8 | 3/8 | 8/8 |
| **Prostate** | Adenocarcinoma | 2/8 | 1/8 | 6/8 |
| **Testis** | Seminoma | 4/8 | 1/8 | 3/8 |
| **Kidney** | Clear cell tumor | 2/8 | 1/8 | 5/8 |
|  | Granular cell tumor | 3/8 | 1/4 | 4/8 |
| **Urinary Blader** | Transitional carcinoma | 5/8 | 2/8 | 1/8 |
