# Supplementary material for: Expression of TAM-R in Human Immune Cells and Unique Regulatory Function of MerTK in IL-10 Production by Tolerogenic DC
Source: Front Immunol. 2020 Sep 25;11:564133. doi: 10.3389/fimmu.2020.564133 (PMC7546251; doi:10.3389/fimmu.2020.564133)
Supplement: Supplementary file 3 [file Data_Sheet_1.PDF]

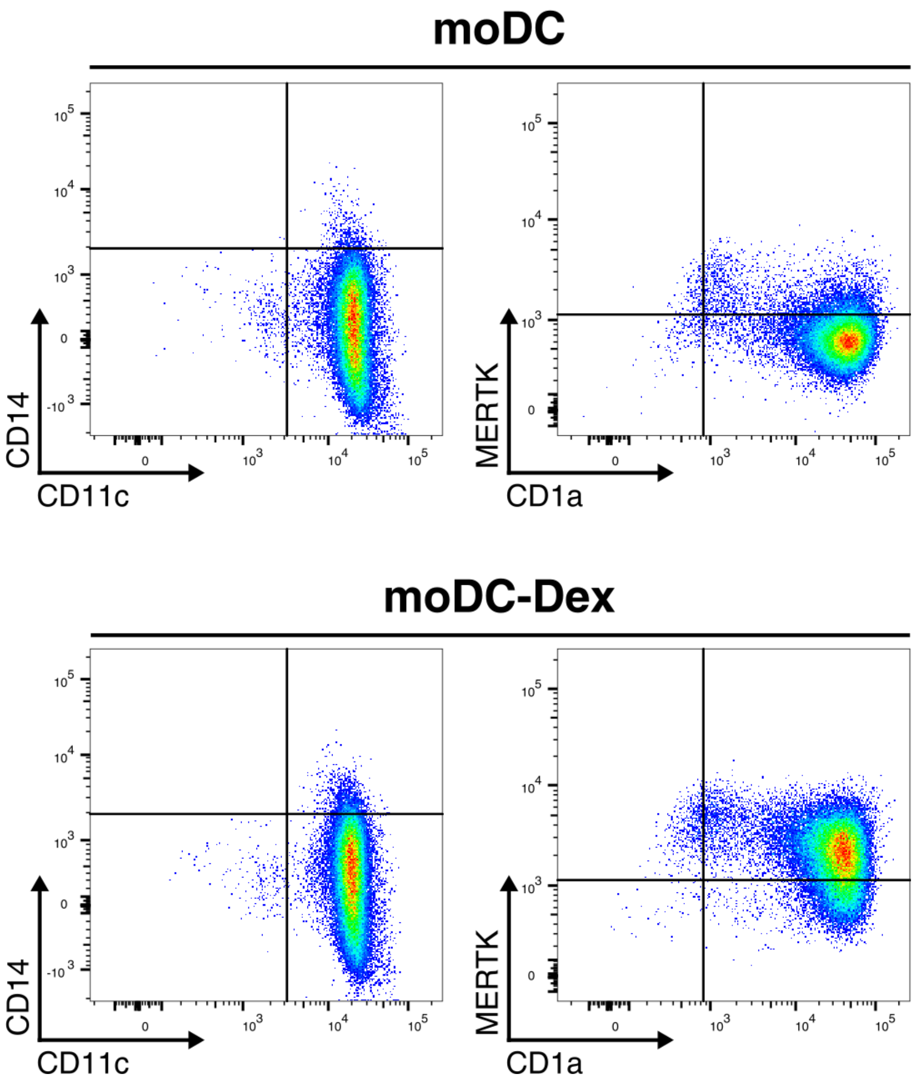

Supplementary Figure 1. Representative dotplots of CD11c, CD14, CD1a and MERTK expression in moDC and moDC-Dex. Quadrants were placed according to relevant isotype control.
