# Supplementary material for: Expression of TAM-R in Human Immune Cells and Unique Regulatory Function of MerTK in IL-10 Production by Tolerogenic DC
Source: Front Immunol. 2020 Sep 25;11:564133. doi: 10.3389/fimmu.2020.564133 (PMC7546251; doi:10.3389/fimmu.2020.564133)
Supplement: Supplementary file 4 [file Data_Sheet_2.PDF]

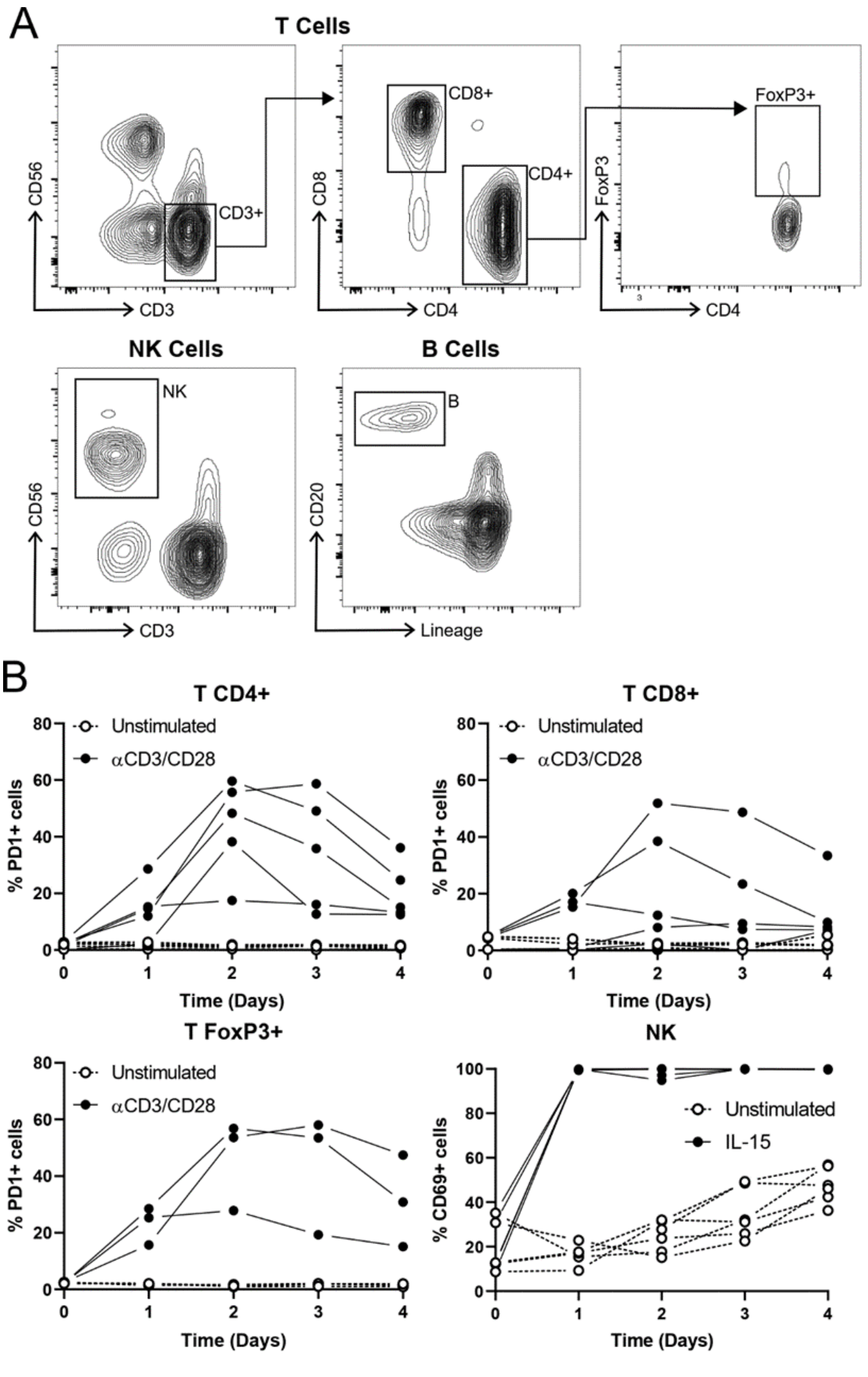

Supplementary Figure 2. (A) Gating strategy used on live peripheral blood cells to identify T $CD4^+$, $CD8^+$, $CD4^+Foxp3^+$, NK and B cells. **(B)** Expression of PD1 on T cells subsets and CD69 on NK cells in resting and activated cells. Full dots represent activated samples and hollow dots represent unstimulated samples, with one dot per donor. T CD4+, CD8+ and NK: n=5. T $CD4^+FoxP3^+$: n=3.
